# Supplementary material for: High-Energy, Whole-Body Proton Irradiation Differentially Alters Long-Term Brain Pathology and Behavior Dependent on Sex and Alzheimer’s Disease Mutations
Source: Int J Mol Sci. 2023 Feb 10;24(4):3615. doi: 10.3390/ijms24043615 (PMC9965515; doi:10.3390/ijms24043615)
Supplement: Supplementary file 1 [file ijms-24-03615-s001.zip › ijms-2190698-supplementary.pdf]

High-Energy, Whole-Body Proton Irradiation Differentially Alters Long-Term Brain Pathology and Behavior Dependent on Sex and Alzheimer's Disease Mutations

Robert G Hinshaw [1,2], Maren K Schroeder [1], Jason Ciola [1], Curran Varma [1], Brianna Colletti [1], Bin Liu [1,3], Grace Geyu Liu [1], Qiaoqiao Shi [1,3], Jacqueline P Williams [4], M Kerry O'Banion [5], Barbara J Caldarone [6], Cynthia A Lemere [1,3]

1: Department of Neurology, Ann Romney Center for Neurologic Diseases, Brigham and Women's Hospital, Boston, MA 02115, USA.
2: Harvard-MIT Division of Health Sciences and Technology, Massachusetts Institute of Technology, Cambridge, MA 02129, USA.
3: Departments of Neurology, Harvard Medical School, Boston, MA 02115, USA.
4: Department of Environmental Medicine, University of Rochester Medical Center, Rochester, NY 14642, USA.
5: Department of Neuroscience, Del Monte Institute of Neuroscience, University of Rochester Medical Center, Rochester, NY 14642, USA.
6: Mouse Behavioral Core, Harvard Medical School, Boston, MA 02115, USA.

Supplemental Methods – SHIRPA

The SmithKline Beecham, Harwell, Imperial College, Royal London Hospital phenotype assessment (SHIRPA) was used to assess overall health and function of mice undergoing behavior tests to screen for gross behavioral outliers[38]. A semiquantitative subset of the full multi-test SHIRPA screen was evaluated for each mouse after a brief observation period, and the scores were combined into an aggregate SHIRPA score. Briefly, mice are observed individually first in a viewing jar and are scored for body position, tremor, palpebral closure, coat appearance, whiskers, lacrimation, and defecation. Then mice are moved to an open arena and scored for transfer arousal, locomotor activity, gait, tail elevation, startle response, touch escape. Lastly, mice are held above the arena and scored for position passivity, skin color, trunk curl, limb grasping, righting reflex, corneal and pineal reflexes, biting, and vocalization.

38. Rogers DC, Fisher EM, Brown SD, Peters J, Hunter AJ, Martin JE. Behavioral and functional analysis of mouse phenotype: SHIRPA, a proposed protocol for comprehensive phenotype assessment. *Mamm Genome.* 1997 Oct;8(10):711-3. doi: 10.1007/s003359900551.

Supplementary Figure S1

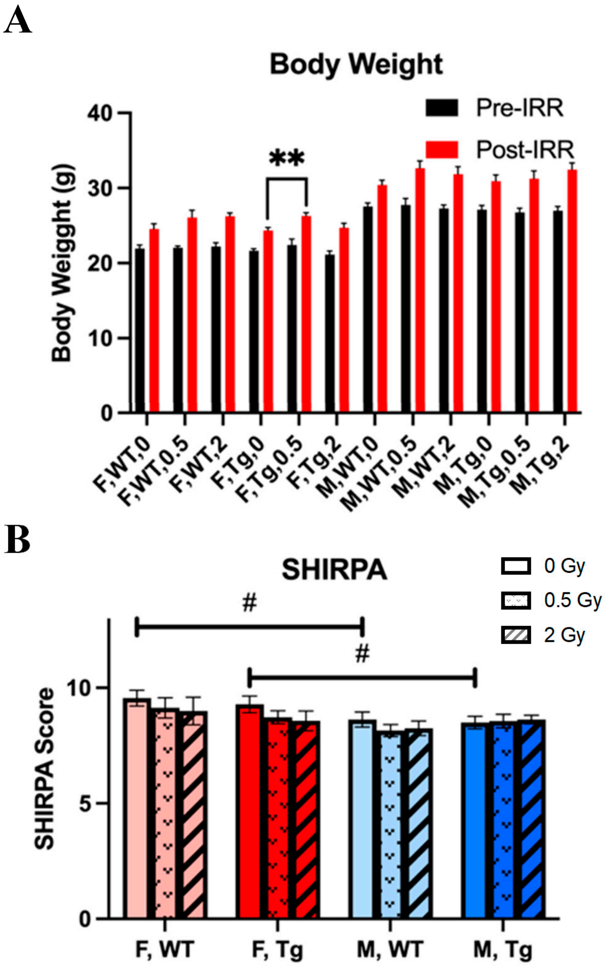

Figure S1: (A) Absolute bodyweight by sex/genotype/dose group at 4 months of age (pre-irradiation) and at 11 months of age (7 months post-irradiation). (**B)** SHIRPA scoring measuring general health. No radiation effects were observed, but female mice trended higher than male mice. (n=6-9 mice/group) (n=10-16 mice/group) #: p<0.1, **: p<0.01. Data were analyzed by 3-way ANOVA followed by post-hoc multiple pairwise comparisons within sex/genotype groups using Tukey's correction. Sham irradiated control groups were compared by planned, 2-tailed t-tests independent of ANOVA.
